# 1-Methyl­piperazine-1,4-dium bis­(hydrogen oxalate)

**DOI:** 10.1107/S1600536814003559

**Published:** 2014-02-22

**Authors:** Manel Essid, Houda Marouani, Mohamed Rzaigui

**Affiliations:** a Laboratoire de Chimie des Matériaux, Faculté des Sciences de Bizerte, 7021 Zarzouna Bizerte, Tunisia

## Abstract

In the crystal structure of the title compound, C_5_H_14_N_2_^2+^·2HC_2_O_4_^−^, the two crystallographically independent hydrogen oxalate anions are linked by strong inter­molecular O—H⋯O hydrogen bonds, forming two independent corrugated chains parallel to the *b* axis. These chains are further connected by N—H⋯O and C—H⋯O hydrogen bonds originating from the organic cations, forming a three-dimensional network. The diprotonated piperazine ring adopts a chair conformation, with the methyl group occupying an equatorial position.

## Related literature   

For the biological activity of piperazines, see: Conrado *et al.* (2008 ▶); Brockunier *et al.* (2004 ▶); Bogatcheva *et al.* (2006 ▶). For related structures, see: Essid *et al.* (2013 ▶); Dutkiewicz *et al.* (2011 ▶); Vaidhyanathan *et al.* (2002 ▶); Ejsmont & Zaleski (2006 ▶). For puckering parameters, see: Cremer & Pople (1975 ▶).
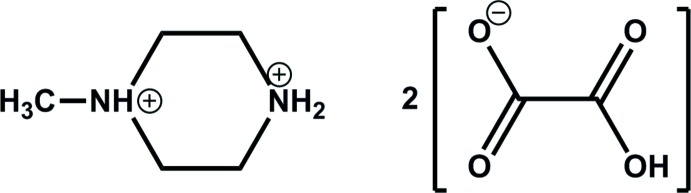


## Experimental   

### 

#### Crystal data  

C_5_H_14_N_2_^2+^·2C_2_HO_4_^−^
*M* *_r_* = 280.24
Monoclinic, 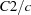

*a* = 15.649 (2) Å
*b* = 5.681 (3) Å
*c* = 27.230 (2) Å
β = 104.05 (2)°
*V* = 2348.4 (13) Å^3^
*Z* = 8
Ag *K*α radiation
λ = 0.56083 Å
μ = 0.08 mm^−1^
*T* = 293 K
0.35 × 0.25 × 0.15 mm

#### Data collection  

Enraf–Nonius CAD-4 diffractometer
7879 measured reflections
5758 independent reflections
3621 reflections with *I* > 2σ(*I*)
*R*_int_ = 0.027
2 standard reflections every 120 min intensity decay: none

#### Refinement  

*R*[*F* ^2^ > 2σ(*F* ^2^)] = 0.052
*wR*(*F* ^2^) = 0.162
*S* = 1.01
5757 reflections
175 parameters
H-atom parameters constrained
Δρ_max_ = 0.41 e Å^−3^
Δρ_min_ = −0.40 e Å^−3^

### 

Data collection: *CAD-4 EXPRESS* (Enraf–Nonius, 1994 ▶); cell refinement: *CAD-4 EXPRESS*; data reduction: *XCAD4* (Harms & Wocadlo, 1995 ▶); program(s) used to solve structure: *SHELXS86* (Sheldrick, 2008 ▶); program(s) used to refine structure: *SHELXL97* (Sheldrick, 2008 ▶); molecular graphics: *ORTEP-3 for Windows* (Farrugia, 2012 ▶) and *DIAMOND* (Brandenburg & Putz, 2005 ▶); software used to prepare material for publication: *WinGX* (Farrugia, 2012 ▶).

## Supplementary Material

Crystal structure: contains datablock(s) I. DOI: 10.1107/S1600536814003559/zl2578sup1.cif

Structure factors: contains datablock(s) I. DOI: 10.1107/S1600536814003559/zl2578Isup2.hkl

CCDC reference: 987380

Additional supporting information:  crystallographic information; 3D view; checkCIF report

## Figures and Tables

**Table 1:** Hydrogen-bond geometry (Å, °)

*D*—H⋯*A*	*D*—H	H⋯*A*	*D*⋯*A*	*D*—H⋯*A*
O2—H2⋯O3^i^	0.82	1.72	2.5242 (17)	167
O5—H5⋯O8^ii^	0.82	1.74	2.5467 (16)	169
N1—H1⋯O4	0.91	1.92	2.7452 (15)	151
N1—H1⋯O2	0.91	2.27	2.9085 (13)	127
N2—H2*C*⋯O8^iii^	0.90	2.03	2.8080 (14)	144
N2—H2*C*⋯O6^iii^	0.90	2.51	3.2564 (19)	141
N2—H2*D*⋯O7^iv^	0.90	1.93	2.7633 (16)	154
N2—H2*D*⋯O5^iv^	0.90	2.32	2.9243 (13)	125
C1—H1*B*⋯O3^v^	0.96	2.45	3.2653 (19)	142
C2—H2*A*⋯O4^i^	0.97	2.44	3.3533 (18)	157
C3—H3*A*⋯O6^vi^	0.97	2.49	3.4334 (18)	163
C3—H3*B*⋯O8^ii^	0.97	2.29	3.2319 (15)	164
C4—H4*B*⋯O7^vii^	0.97	2.43	3.3665 (18)	163
C5—H5*A*⋯O3^viii^	0.97	2.28	3.2269 (16)	165

Symmetry codes: (i) 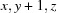; (ii) 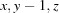; (iii) 
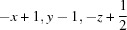
; (iv) 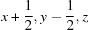; (v) 
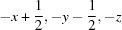
; (vi) 
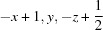
; (vii) 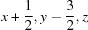; (viii) 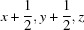.

**Fig. 1.**
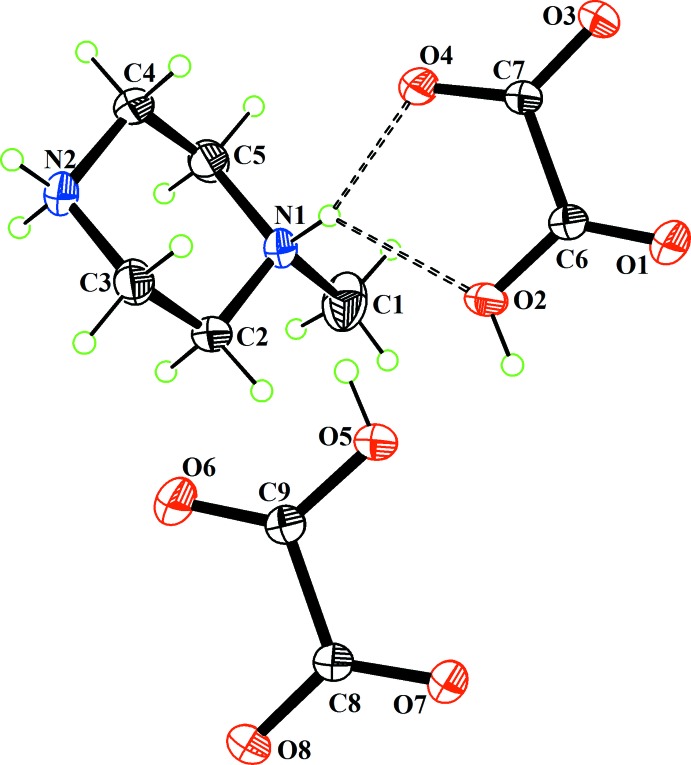
An ORTEP view of (I) with the atom-labelling scheme. Displacement ellipsoids are drawn at the 45% probability level. H atoms are represented as small spheres of arbitrary radii.

**Fig. 2.**
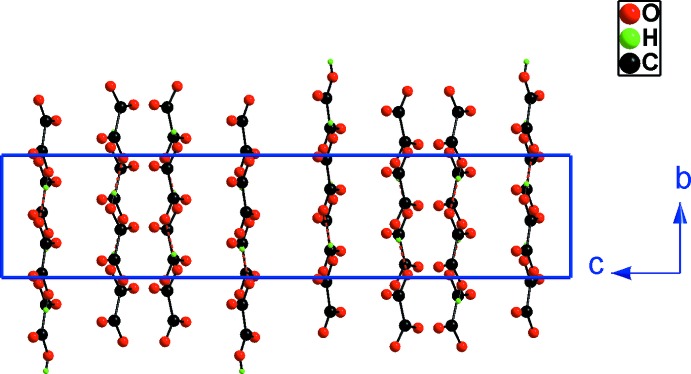
Projection of the corrugated hydrogen oxalate chains along the a axis.

**Fig. 3.**
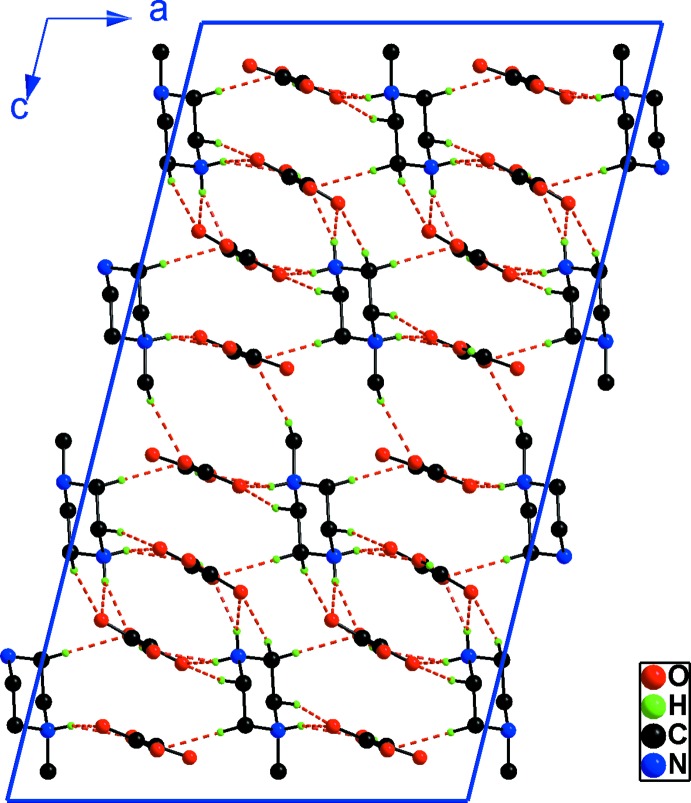
Projection of (I) along the b axis. The H-atoms not involved in H-bonding are omitted.

## supplementary crystallographic information

### 1. Comment

Piperazine and its derivatives have been intensively investigated owing to their interesting pharmacological, cardiovascular and autonomic properties (Conrado *et al.*, 2008). Piperazine derivatives are found in biologically active compounds across a number of different therapeutic areas such as antifungal, antibacterial, antimalarial, antipsychotic, antidepressant and antitumour activity against colon, prostate, breast, lung and leukemia tumors (Brockunier *et al.*, 2004; Bogatcheva *et al.*, 2006; Essid *et al.*, 2013). In the present work, we report the preparation and the crystal structure of an organic proton transfer salt (C_5_H_14_N_2_)^2+^·2(HC_2_O_4_)^-^, (I). The asymmetric unit of (I) contains one 1-methylpiperazin-1,4-diium dication and two semi-oxalate anions (Fig. 1). 1-Methylpiperazine is diprotonated at atom N1 and N2 and oxalic acid is mono-deprotonated. The oxalate monoanions are essentially planar, with dihedral angles between the carboxylate groups of less than 4°. Two strong O–H···O (Table 1) hydrogen bonds generate linear oxalate chains running parallel to the *b* axis (Fig. 2). The geometrical parameters of these chains correlate well with the corresponding values found in related crystal structures (Essid *et al.*, 2013; Vaidhyanathan *et al.*, 2002; Ejsmont & Zaleski, 2006). Bond distances around atom C7 and C8 indicate a carboxylate group with delocalization of the negative charge between atoms O3 and O4, and between O7 and O8. In the hydrogenoxalate anion HC_2_O_4_^-^, the H atoms are located at O2 and O5. The position of protonation is also indicated by elongation of the corresponding C–O distances [O2–C6 = 1.306 (2) Å, O5–C9 = 1.306 (1) Å]. The bond lengths of C6–C7 and C8–C9 are relatively long [1.553 (2) Å, 1.544 (2) Å] as expected for an oxalate anion. Geometrical parameters of the methylpiperazin-1,4-dium dications are found to be in agreement with those of another similar structure of methylpiperazin-1,4-diium dipicrate (Dutkiewicz *et al.*, 2011). The cyclic amine adopts a chair conformation with the methyl group occupying an equatorial position, with puckering parameters: Q = 0.5772 (11) A, θ = 2.85 (11)° and φ = -174 (2)° (Cremer & Pople, 1975) and atoms N1 and N2 deviating by -0.308 (2) and 0.333 (2) Å from the least-squares plane defined by the remaining atoms in the ring. In addition, the crystal structure of [C_5_H_14_N_2_](HC_2_O_4_)_2_ is stabilized by ionic interactions between the 1-methylpiperazin-1,4-dium dications and the oxalate monoanions chains, as well as by a network of N–H···O and C–H···O hydrogen bonds (Fig. 3 and Table 1) such that all the hydrogen atoms bonded to nitrogen atoms participate in the formation of these hydrogen bonds, with donor-acceptor distances between 2.745 (2) and 3.433 (2) Å (Table 1).

### 2. Experimental

An aqueous solution containing 2 mmol of H_2_C_2_O_4_ in 20 ml of water was added to 1 mmol of 1-methylpiperazine in 10 ml of ethanol. The obtained solution was stirred at 333 K. When the solution became homogeneous it was cooled slowly and kept at room temperature. After several days, transparent colourless crystals formed. Crystals of the title compound, which remained stable under normal conditions of temperature and humidity, were isolated and subjected to X-ray diffraction analysis. M.p. 260°C. Main IR bands (KBr disc, cm^-1^): (vs = very strong; s = strong; w = weak) 3025 w, 1619 s, 1470 s, 1410 s, 1356 vs, 1269 vs, 1203 w, 1050 vs, 1022 s, 985 s, 713 s.

### 3. Refinement

All H atoms were located in a difference map. Nevertheless, they were geometrically placed and refined using a riding model, with C—H = 0.96 Å (methyl) or 0.97 Å (methylene), N—H = 0.90 Å or 0.91 Å and O—H = 0.82 Å with *U*_iso_(H) = 1.2*U*_eq_(C or N) or 1.5*U*_eq_(O).

### Crystal data

**Table:**

C_5_H_14_N_2_^2+^·2C_2_HO_4_^−^	*F*(000) = 1184
*M**_r_* = 280.24	*D*_x_ = 1.585 Mg m^−^^3^
Monoclinic, *C*2/*c*	Ag *K*α radiation, λ = 0.56083 Å
Hall symbol: -C 2yc	Cell parameters from 25 reflections
*a* = 15.649 (2) Å	θ = 9–11°
*b* = 5.681 (3) Å	µ = 0.08 mm^−^^1^
*c* = 27.230 (2) Å	*T* = 293 K
β = 104.05 (2)°	Prism, colourless
*V* = 2348.4 (13) Å^3^	0.35 × 0.25 × 0.15 mm
*Z* = 8	

### Data collection

**Table:**

Enraf–Nonius CAD-4 diffractometer	*R*_int_ = 0.027
Radiation source: fine-focus sealed tube	θ_max_ = 28.0°, θ_min_ = 2.1°
Graphite monochromator	*h* = −26→25
non–profiled ω scans	*k* = −2→9
7879 measured reflections	*l* = −1→45
5758 independent reflections	2 standard reflections every 120 min
3621 reflections with *I* > 2σ(*I*)	intensity decay: none

### Refinement

**Table:**

Refinement on *F*^2^	Primary atom site location: structure-invariant direct methods
Least-squares matrix: full	Secondary atom site location: difference Fourier map
*R*[*F*^2^ > 2σ(*F*^2^)] = 0.052	Hydrogen site location: inferred from neighbouring sites
*wR*(*F*^2^) = 0.162	H-atom parameters constrained
*S* = 1.01	*w* = 1/[σ^2^(*F*_o_^2^) + (0.0887*P*)^2^ + 0.6525*P*] where *P* = (*F*_o_^2^ + 2*F*_c_^2^)/3
5757 reflections	(Δ/σ)_max_ = 0.006
175 parameters	Δρ_max_ = 0.41 e Å^−^^3^
0 restraints	Δρ_min_ = −0.40 e Å^−^^3^

### Special details

**Table:**

Geometry. All e.s.d.'s (except the e.s.d. in the dihedral angle between two l.s. planes) are estimated using the full covariance matrix. The cell e.s.d.'s are taken into account individually in the estimation of e.s.d.'s in distances, angles and torsion angles; correlations between e.s.d.'s in cell parameters are only used when they are defined by crystal symmetry. An approximate (isotropic) treatment of cell e.s.d.'s is used for estimating e.s.d.'s involving l.s. planes.
Refinement. Refinement of *F*^2^ against ALL reflections. The weighted *R*-factor *wR* and goodness of fit *S* are based on *F*^2^, conventional *R*-factors *R* are based on *F*, with *F* set to zero for negative *F*^2^. The threshold expression of *F*^2^ > σ(*F*^2^) is used only for calculating *R*-factors(gt) *etc*. and is not relevant to the choice of reflections for refinement. *R*-factors based on *F*^2^ are statistically about twice as large as those based on *F*, and *R*- factors based on ALL data will be even larger.

### Fractional atomic coordinates and isotropic or equivalent isotropic displacement parameters (Å^2^)

**Table:**

	*x*	*y*	*z*	*U*_iso_*/*U*_eq_	
O5	0.26348 (5)	0.43588 (15)	0.19137 (4)	0.02858 (19)	
H5	0.2892	0.3099	0.1980	0.043*	
O6	0.39346 (6)	0.58784 (19)	0.23195 (5)	0.0436 (3)	
O7	0.19490 (5)	0.86264 (16)	0.17619 (4)	0.02856 (19)	
O8	0.32494 (5)	1.02361 (15)	0.21317 (4)	0.02746 (18)	
C8	0.27389 (6)	0.85255 (19)	0.19799 (4)	0.02018 (18)	
C9	0.31747 (7)	0.60820 (19)	0.20900 (4)	0.02266 (19)	
O1	0.12751 (6)	−0.01254 (18)	0.05548 (4)	0.0373 (2)	
O2	0.26291 (6)	0.13819 (16)	0.08184 (4)	0.0348 (2)	
H2	0.2366	0.2642	0.0780	0.052*	
O3	0.20184 (6)	−0.45041 (16)	0.06515 (4)	0.0331 (2)	
O4	0.33311 (5)	−0.28288 (16)	0.09557 (4)	0.0334 (2)	
C6	0.20627 (7)	−0.03411 (19)	0.07029 (5)	0.0231 (2)	
C7	0.25214 (7)	−0.27840 (19)	0.07768 (4)	0.02206 (19)	
N1	0.45019 (6)	0.07065 (18)	0.09040 (4)	0.02256 (18)	
H1	0.4022	−0.0041	0.0963	0.027*	
N2	0.57669 (6)	0.01455 (19)	0.18579 (4)	0.0264 (2)	
H2C	0.5933	−0.0381	0.2179	0.032*	
H2D	0.6228	0.0897	0.1786	0.032*	
C1	0.42704 (10)	0.1657 (3)	0.03769 (5)	0.0418 (3)	
H1A	0.3819	0.2830	0.0347	0.063*	
H1B	0.4060	0.0401	0.0143	0.063*	
H1C	0.4783	0.2351	0.0302	0.063*	
C2	0.47453 (7)	0.2672 (2)	0.12710 (5)	0.0261 (2)	
H2A	0.4245	0.3722	0.1237	0.031*	
H2B	0.5225	0.3562	0.1193	0.031*	
C3	0.50225 (7)	0.1799 (2)	0.18088 (5)	0.0270 (2)	
H3A	0.5198	0.3118	0.2037	0.032*	
H3B	0.4532	0.1011	0.1899	0.032*	
C4	0.55098 (8)	−0.1872 (2)	0.15092 (5)	0.0298 (2)	
H4A	0.5024	−0.2710	0.1593	0.036*	
H4B	0.6002	−0.2952	0.1547	0.036*	
C5	0.52395 (7)	−0.1014 (2)	0.09704 (5)	0.0284 (2)	
H5A	0.5739	−0.0274	0.0881	0.034*	
H5B	0.5057	−0.2342	0.0746	0.034*	

### Atomic displacement parameters (Å^2^)

**Table:**

	*U*^11^	*U*^22^	*U*^33^	*U*^12^	*U*^13^	*U*^23^
O5	0.0224 (3)	0.0150 (3)	0.0441 (5)	0.0008 (3)	−0.0001 (3)	−0.0021 (3)
O6	0.0219 (4)	0.0272 (5)	0.0706 (7)	0.0024 (3)	−0.0106 (4)	0.0053 (5)
O7	0.0187 (3)	0.0206 (4)	0.0417 (5)	0.0014 (3)	−0.0019 (3)	0.0026 (3)
O8	0.0244 (4)	0.0164 (3)	0.0378 (5)	−0.0035 (3)	0.0001 (3)	−0.0013 (3)
C8	0.0195 (4)	0.0160 (4)	0.0237 (4)	0.0000 (3)	0.0026 (3)	0.0004 (3)
C9	0.0187 (4)	0.0172 (4)	0.0299 (5)	0.0003 (3)	0.0016 (3)	0.0008 (4)
O1	0.0189 (4)	0.0270 (5)	0.0608 (6)	0.0032 (3)	−0.0003 (4)	0.0064 (4)
O2	0.0218 (4)	0.0144 (3)	0.0647 (6)	−0.0003 (3)	0.0039 (4)	−0.0006 (4)
O3	0.0243 (4)	0.0166 (4)	0.0532 (6)	−0.0030 (3)	−0.0006 (4)	−0.0024 (4)
O4	0.0180 (3)	0.0194 (4)	0.0588 (6)	0.0012 (3)	0.0011 (3)	0.0037 (4)
C6	0.0204 (4)	0.0166 (4)	0.0308 (5)	0.0008 (3)	0.0032 (4)	0.0021 (4)
C7	0.0196 (4)	0.0146 (4)	0.0304 (5)	−0.0001 (3)	0.0029 (3)	0.0002 (4)
N1	0.0172 (3)	0.0229 (4)	0.0255 (4)	−0.0018 (3)	0.0012 (3)	0.0008 (3)
N2	0.0183 (4)	0.0270 (5)	0.0298 (5)	−0.0009 (3)	−0.0022 (3)	0.0022 (4)
C1	0.0363 (6)	0.0563 (10)	0.0291 (6)	−0.0029 (7)	0.0009 (5)	0.0108 (6)
C2	0.0234 (4)	0.0174 (4)	0.0347 (6)	0.0014 (4)	0.0018 (4)	−0.0006 (4)
C3	0.0220 (4)	0.0276 (5)	0.0298 (5)	0.0005 (4)	0.0034 (4)	−0.0054 (4)
C4	0.0229 (5)	0.0185 (5)	0.0437 (7)	0.0031 (4)	−0.0006 (4)	0.0006 (5)
C5	0.0221 (4)	0.0259 (5)	0.0367 (6)	0.0013 (4)	0.0061 (4)	−0.0075 (5)

### Geometric parameters (Å, º)

**Table:**

O5—C9	1.3061 (14)	N2—C4	1.4805 (17)
O5—H5	0.8200	N2—H2C	0.9000
O6—C9	1.2070 (13)	N2—H2D	0.9000
O7—C8	1.2358 (12)	C1—H1A	0.9600
O8—C8	1.2622 (13)	C1—H1B	0.9600
C8—C9	1.5437 (16)	C1—H1C	0.9600
O1—C6	1.2063 (13)	C2—C3	1.5068 (18)
O2—C6	1.3067 (14)	C2—H2A	0.9700
O2—H2	0.8200	C2—H2B	0.9700
O3—C7	1.2493 (14)	C3—H3A	0.9700
O4—C7	1.2424 (13)	C3—H3B	0.9700
C6—C7	1.5530 (16)	C4—C5	1.5059 (19)
N1—C2	1.4854 (16)	C4—H4A	0.9700
N1—C5	1.4897 (15)	C4—H4B	0.9700
N1—C1	1.4934 (17)	C5—H5A	0.9700
N1—H1	0.9100	C5—H5B	0.9700
N2—C3	1.4770 (15)		
			
C9—O5—H5	109.5	H1A—C1—H1B	109.5
O7—C8—O8	126.96 (10)	N1—C1—H1C	109.5
O7—C8—C9	118.57 (9)	H1A—C1—H1C	109.5
O8—C8—C9	114.46 (9)	H1B—C1—H1C	109.5
O6—C9—O5	125.91 (11)	N1—C2—C3	111.87 (10)
O6—C9—C8	121.33 (10)	N1—C2—H2A	109.2
O5—C9—C8	112.75 (9)	C3—C2—H2A	109.2
C6—O2—H2	109.5	N1—C2—H2B	109.2
O1—C6—O2	125.63 (11)	C3—C2—H2B	109.2
O1—C6—C7	122.46 (10)	H2A—C2—H2B	107.9
O2—C6—C7	111.90 (9)	N2—C3—C2	109.40 (10)
O4—C7—O3	127.31 (10)	N2—C3—H3A	109.8
O4—C7—C6	117.67 (9)	C2—C3—H3A	109.8
O3—C7—C6	115.01 (9)	N2—C3—H3B	109.8
C2—N1—C5	110.32 (9)	C2—C3—H3B	109.8
C2—N1—C1	109.73 (11)	H3A—C3—H3B	108.2
C5—N1—C1	110.72 (11)	N2—C4—C5	110.04 (10)
C2—N1—H1	108.7	N2—C4—H4A	109.7
C5—N1—H1	108.7	C5—C4—H4A	109.7
C1—N1—H1	108.7	N2—C4—H4B	109.7
C3—N2—C4	110.41 (9)	C5—C4—H4B	109.7
C3—N2—H2C	109.6	H4A—C4—H4B	108.2
C4—N2—H2C	109.6	N1—C5—C4	110.90 (10)
C3—N2—H2D	109.6	N1—C5—H5A	109.5
C4—N2—H2D	109.6	C4—C5—H5A	109.5
H2C—N2—H2D	108.1	N1—C5—H5B	109.5
N1—C1—H1A	109.5	C4—C5—H5B	109.5
N1—C1—H1B	109.5	H5A—C5—H5B	108.0

### Hydrogen-bond geometry (Å, º)

**Table:**

*D*—H···*A*	*D*—H	H···*A*	*D*···*A*	*D*—H···*A*
O2—H2···O3^i^	0.82	1.72	2.5242 (17)	167
O5—H5···O8^ii^	0.82	1.74	2.5467 (16)	169
N1—H1···O4	0.91	1.92	2.7452 (15)	151
N1—H1···O2	0.91	2.27	2.9085 (13)	127
N2—H2*C*···O8^iii^	0.90	2.03	2.8080 (14)	144
N2—H2*C*···O6^iii^	0.90	2.51	3.2564 (19)	141
N2—H2*D*···O7^iv^	0.90	1.93	2.7633 (16)	154
N2—H2*D*···O5^iv^	0.90	2.32	2.9243 (13)	125
C1—H1*B*···O3^v^	0.96	2.45	3.2653 (19)	142
C2—H2*A*···O4^i^	0.97	2.44	3.3533 (18)	157
C3—H3*A*···O6^vi^	0.97	2.49	3.4334 (18)	163
C3—H3*B*···O8^ii^	0.97	2.29	3.2319 (15)	164
C4—H4*B*···O7^vii^	0.97	2.43	3.3665 (18)	163
C5—H5*A*···O3^viii^	0.97	2.28	3.2269 (16)	165

Symmetry codes: (i) *x*, *y*+1, *z*; (ii) *x*, *y*−1, *z*; (iii) −*x*+1, *y*−1, −*z*+1/2; (iv) *x*+1/2, *y*−1/2, *z*; (v) −*x*+1/2, −*y*−1/2, −*z*; (vi) −*x*+1, *y*, −*z*+1/2; (vii) *x*+1/2, *y*−3/2, *z*; (viii) *x*+1/2, *y*+1/2, *z*.
